# Drought Stress Might Induce Sexual Spatial Segregation in Dioecious *Populus euphratica*—Insights from Long-Term Water Use Efficiency and Growth Rates

**DOI:** 10.3390/biology13050318

**Published:** 2024-05-02

**Authors:** Honghua Zhou, Zhaoxia Ye, Yuhai Yang, Chenggang Zhu

**Affiliations:** State Key Laboratory of Desert and Oasis Ecology, Xinjiang Institute of Ecology and Geography, Chinese Academy of Sciences, Urumqi 830011, China; zhouhh@ms.xjb.ac.cn (H.Z.); yezx@ms.xjb.ac.cn (Z.Y.); yangyh@ms.xjb.ac.cn (Y.Y.)

**Keywords:** ecological water conveyance, groundwater depth change, annual growth rate, intrinsic water use efficiency, lower Tarim River

## Abstract

**Simple Summary:**

Dioecious plants hold an important position in maintaining species diversity and the structural and functional stability of terrestrial plant ecosystems. *Populus euphratica* (*P. euphratica*), a prototypical dioecious tree with a global distribution across desert and arid regions, is a notable model plant for investigating mechanisms of environmental stress resistance. In this study, we studied the responses of the sex ratio, age structure, survival curve, growth rate, and WUE_i_ exhibited by male and female *P. euphratica* in the lower Tarim River to change groundwater depth. We found that the drought tolerance range of males was narrower than that of females; moreover, male *P. euphratica* were more competitive under mild drought stress, while female *P. euphratica* demonstrated greater endurance under severe drought stress. These findings elucidated the reason behind gender-based spatial differentiation in *P. euphratica* from the perspective of specialized water use between males and females and shed new light on the implications of ecological water conveyance management for the restoration, conservation, and rejuvenation of the natural *P. euphratica* forest.

**Abstract:**

*P. euphratica* stands as the pioneering and dominant tree within desert riparian forests in arid and semi-arid regions. The aim of our work was to reveal why dioecious *P. euphratica* in natural desert riparian forests in the lower Tarim River exhibits sexual spatial distribution differences combined with field investigation, tree ring techniques, isotope analysis techniques, and statistical analyses. The results showed that *P. euphratica* was a male-biased population, with the operational sex ratio (OSR) exhibiting spatial distribution differences to variations in drought stress resulting from groundwater depth change. The highest OSR was observed under mild drought stress (groundwater depth of 6–7 m), and it was reduced under non-drought stress (groundwater depth below 6 m) or severe drought stress (groundwater depth exceeding 7 m). As drought stress escalated, the degradation and aging of the *P. euphratica* forest became more pronounced. Males exhibited significantly higher growth rates and *WUE_i_* than females under mild drought stress. However, under severe drought stress, males’ growth rates significantly slowed down, accompanied by significantly lower *WUE_i_* than in females. This divergence determined the sexual spatial segregation of *P. euphratica* in the natural desert riparian forests of the lower Tarim River. Furthermore, the current ecological water conveyance project (EWCP) in the lower Tarim River was hard to fundamentally reverse the degradation and aging of the *P. euphratica* forest due to inadequate population regeneration. Consequently, we advocated for an optimized ecological water conveyance mode to restore, conserve, and rejuvenate natural *P. euphratica* forests.

## 1. Introduction

A total of 15,600 angiosperms exhibited dioecious traits in their natural state, constituting approximately 5–6% of the total species, 7% of genera, and 43% of families [1]. Within terrestrial plant ecosystems, dioecious plants hold an important position, contributing substantially to the preservation of species diversity and the maintenance of structural and functional stability in these ecosystems [2]. Typically, females and males display distinct responses to shifts in the environment. Numerous studies have indicated that males perform better than females in growth and morphology, photosynthetic capacity, resource use efficiency, and resistance under abiotic environmental stresses because females need a higher reproduction investment to produce flowers and seeds [2,3,4,5]. Consequently, environmental factors possess the potential to alter the sex ratio of dioecious plants, consequently influencing their sexual spatial segregation patterns along abiotic gradients.

Among abiotic environmental stresses, aridity is a pivotal limiting factor for plant survival, growth, development, and sex allocation [1,6,7,8,9]. Dioecious plants are commonly observed in drier habits, especially in areas where water has become scarce or is in limited supply [9]. With rising temperatures, drought has constituted an increasingly urgent global problem. This intensifying drought has significantly impacted the sex allocation and population dynamics of dioecious plants, ultimately resulting in male- or female-biased distributions [7,10]. Consequently, it is imperative to comprehensively understand sex-related physiological responses to drought stress. This understanding is critical for elucidating sexual spatial differentiation, predicting the population structure, and anticipating the succession patterns of dioecious plants in an increasingly global drought in the future [7,10,11].

Intrinsic water use efficiency (*WUE_i_*), a measure reflecting a plant’s water utilization situation and adaptation to prolonged drought stress, is the most fundamental and pivotal physiological response to ensure the survival and growth of plants under drought stress [12]. *WUE_i_* addresses the limitations of instantaneous water use efficiency (*WUEt*), as the latter only depicts the water usage status of specific plant organs at particular moments, and it struggles to capture the comprehensive, long-term water utilization of the entire plant [13]. To date, *WUE_i_* remains one of the most effective methods for investigating a plant’s long-term water use efficiency [14], and it is extensively employed in the examination of divergent water utilization strategies between male and female plants experiencing environmental stress [4,15,16].

*Populus euphratica* (*P. euphratica*), a notable model plant for investigating the mechanisms of environmental stress resistance [17], stands as a prototypical dioecious tree with a global distribution across desert and arid regions, including northwestern Africa, western South Asia, and Central Asia [18,19]. Serving as an invaluable tree species for desert riparian forests in arid regions, *P. euphratica* assumes a critical role in maintaining ecosystems, preventing wind and sand movement, safeguarding biodiversity, regulating climate, and protecting oases [17,19,20]. The riparian forest along the Tarim River represents the largest and most important *P. euphratica* gene pool worldwide holds a total *P. euphratica* area accounting for approximately 55% globally and over 90% within China [2,21]. This locale offers an ideal setting to explore the sex-specific physiological responses of *P. euphratica* to drought stress in a natural environment [22].

Since the 1970s, due to the lack of precipitation and over-exploitation of surface water resources, the *P. euphratica* forest in the Tarim River Basin has been subject to different drought stresses [23]. Moreover, the Tarim River Basin is one of the most climate-sensitive areas globally [24], making it prone to occur with more frequent and extreme droughts in response to ongoing global warming and groundwater depth fluctuations [25]. Consequently, considerable attention has been directed toward understanding the physiological responses of *P. euphratica* to changing drought stresses in the Tarim River Basin, which encompass aspects such as biomass, cellular microstructure, chlorophyll concentration, net photosynthetic rate, gas exchange, chlorophyll fluorescence, water potential, osmotic adjustment solutes, enzymatic activity, hydraulic conductivity, and uplift, as well as WUEt [24,26,27,28,29]. However, these studies have yet to explore the sex-specific physiological responses of male and female *P. euphratica*.

Studies have indicated that *P. euphratica* exhibits sexual spatial differentiation with a male bias, a variation related closely to changes in groundwater depth [30]. Despite these findings, the underlying causes for the gender-based spatial distribution and sex ratio in their natural settings remain unclear. So far, while a few studies have reported the sex-specific physiological responses of *P. euphratica* seedlings to drought or salt stress through controlled laboratory simulation experiments [2,12,18], limited information exists concerning the sex-specific physiological responses of natural *P. euphratica* to drought stress triggered by fluctuations in groundwater depth, particularly in relation to sex-specific water use efficiency over extended periods. Hence, establishing whether natural *P. euphratica* demonstrates sex-specific responses in *WUE_i_* to groundwater depth changes is of the utmost significance. Such insights are poised to significantly impact the tree growth, population structure, and community succession of *P. euphratica* amidst future climate change characterized by heightened drought occurrences.

Given that the *P. euphratica* forest in the lower Tarim River underwent significantly higher changes in groundwater depths between 1975 and 2015, our study focuses on exploring the corresponding responses of the sex ratio, age structure, survival curve, growth rate, and *WUE_i_* exhibited by male and female *P. euphratica* in the lower Tarim River on fluctuations in groundwater depth. Our primary hypotheses are as follows: (1) does dioecious *P. euphratica* exhibit sex-specific growth rates and *WUE_i_* in response to drought stress triggered by changes in groundwater depth? (2) Could the sex-specific growth rates and *WUE_i_* elucidate the underlying reasons behind the sexual spatial separation observed between male and female *P. euphratica* in the lower Tarim River?

## 2. Materials and Methods

### 2.1. Study Area

The Tarim River, one of the longest arid inland rivers in China, stretches for 1321 km through the Taklimakan and Kuluke deserts in northwestern China, within the mid-latitudes of Eurasia. The Tarim River basin is a closed catchment area in an extremely arid region characterized by a continental warm temperate climate, notable dryness, and pronounced evaporation. Annual precipitation averages between 17.4 and 42.0 mm, while annual potential evaporation averages from approximately 2500 to 3000 mm, which is nearly 60 times that of annual precipitation. Vegetation primarily follows the river, shaping a desert riparian forest. *P. euphratica* is the dominant plant of the desert riparian forest. The growth and development of the desert riparian *P. euphratica* forest hinges upon runoff and groundwater. However, the lower river length of 321 km has been cut-off since the 1970s due to the continuous increase in water consumption in the upper and middle rivers to satisfy cultivated land expansion. The cessation of water supply in the lower reaches forced the *P. euphratica* forest to rely solely on groundwater. After the cut-off, groundwater depth along the lower reaches increased markedly from 3 to 5 m in the 1950s to 8–12 m in the 1990s [31]. These changes in groundwater depth ushered in varying levels of drought stress for the riparian *P. euphratica* forest, ultimately culminating in significant degradation in the lower Tarim River’s *P. euphratica* forest [29]. Furthermore, the *P. euphratica* forest’s width alongside the river channel dwindled, contracting to a mere 1000 m and becoming densely concentrated within 500 m of the river. To conserve and restore the declining desert riparian *P. euphratica* forest, the Chinese government initiated the Ecological Water Conveyance Project (EWCP) in 2000. This project aimed to intermittently supply ecological water from the Daxihaizi Reservoir and Bosten Lake, traversing downstream to decrease groundwater depth. The cut-off and EWCP of the lower river significantly transformed the groundwater environment, consequently altering the drought stress degree of the *P. euphratica* forest. This provided an opportune circumstance for investigating the sex-specific *WUE_i_* of male and female *P. euphratica* under varying drought stress conditions, discussing the development and succession trends of the *P. euphratica* forest under drought stress.

**Figure 1:**
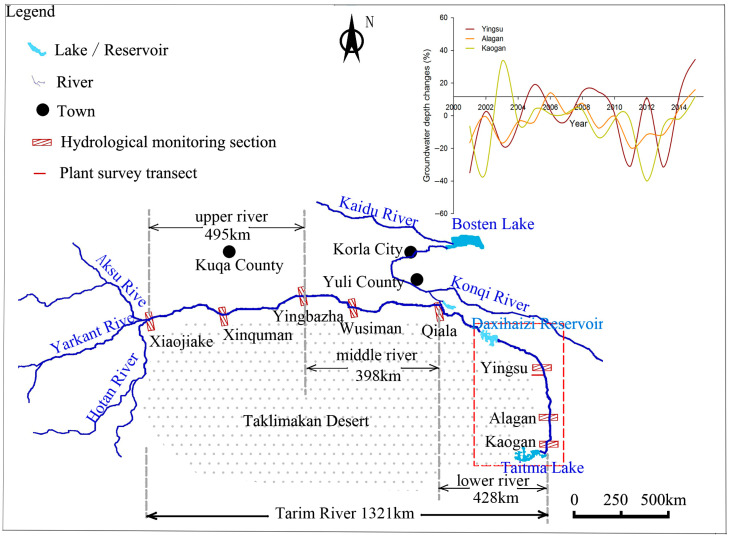
Sketch map of the study area.

To monitor the response of groundwater depths in the lower reaches of the EWCP, three hydrological monitoring sections (Yinsu, Alagan, and Kaogan) were established in the lower reaches starting in 2000. Prior to 2000, the existing *P. euphratica* forests in the lower reaches were predominantly concentrated in the Yingsu section and the Alagan section, with negligible presence in the Kaogan section [32]. Furthermore, the Yingsu section exhibited a more favorable number, density, and growth status of *P. euphratica* than the Alagan section [33]. According to hydrological data from the Tarim River Basin Water Resources Management Bureau, the total discharged water volume from 2000 to 2015 amounted to 51.1 × 10^8^ m^3^. Due to the varying timing and volume of discharged water from the upper river each year, the location where ecological water arrived fluctuated annually [22]. Nonetheless, the Yingsu section consistently received ecological water conveyance and was the area most significantly affected by groundwater fluctuations between 2000 and 2015 (Figure 1). The effective range of influence of EWCP on groundwater depth was less than 1000 m from the vertical distance of the river, in which notable changes in groundwater depth were observed within 500 m from the river [34]. Moreover, the Yingsu section exhibited the most significant changes in *P. euphratica* growth and the highest restorable rates of species diversity following the implementation of the EWCP [35]. Given these factors, the study site was situated within the Yingsu section, approximately 1000 m from the vertical river channel (Figure 1). This location boasts relatively uniform soil texture and consistent meteorological conditions with relatively homogeneous plant species and relatively flat terrain. The Yingsu section is located about 40 km southeast of the Daxihaizi Reservoir, and the surface soil (0–30 cm) in the Yingsu section constitutes a typical desert soil characterized by limited water-holding capacity.

### 2.2. Collection of Groundwater Depth Data

**Figure 2:**
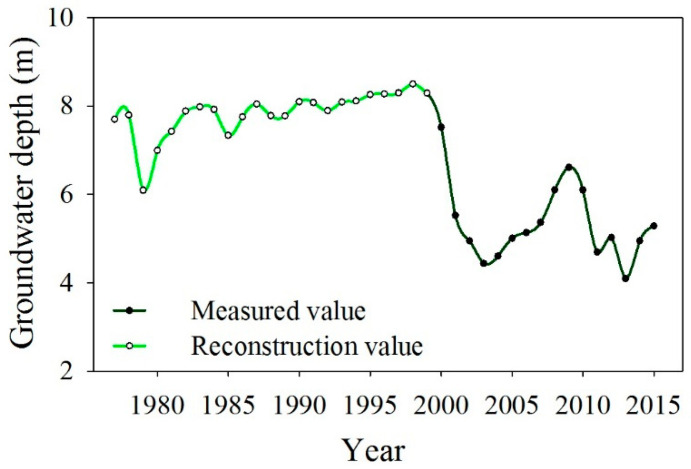
Change in groundwater depth in the study area from 1975 to 2015.

Within the Yingsu hydrosection, six groundwater wells (8–17 m depth) were installed at 50, 150, 250, 500, 750 and 1000 m along the river, respectively. Groundwater depths were monitored every two months starting in 2000. Consequently, the annual average groundwater depth data for the study period of 2000–2015 were derived from the monitoring of groundwater wells in the Yingsu section. However, no groundwater depth measurement data are available before 2000. As a result, researchers reconstructed the annual average groundwater depth data for the Yingsu section from 1976 to 1999 using the tree ring width of *P. euphratica*, and the reconstructed data tracked the major hydrological events in the study area [22,30]. In this study, the reconstructed groundwater depth data from 1976 to 1999 and the measured groundwater depth data from 2000 to 2015 were utilized to establish an annual average groundwater depth data series for the Yingsu section spanning from 1976 to 2015 (Figure 2).

### 2.3. Investigation of Population Structure, Spatial Distribution, and Sex Ratio of P. euphratica

Considering the variation in groundwater depth with the increasing distance from the river channel and aiming to comprehensively investigate the sex ratio and growth patterns of *P. euphratica* in the Yingsu section, we established a plant survey transect measuring 50 m in width (parallel to the river channel) and 1000 m in length (perpendicular to the river channel) around the groundwater monitoring wells within the Yingsu section. The field investigation was conducted during the flowering stage of *P. euphratica* in March 2015. We recorded the quantities, sexes, and diameters at breast height (DBH) of all *P. euphratica* trees within the transect. Male and female trees were judged by the differences in flower morphology. Utilizing common classification standards and developmental characteristics of trees, the age structure of *P. euphratica* was categorized into young trees (DBH ≤ 10cm), half-mature trees (10 < DBH ≤ 25 cm), mature trees (25 < DBH ≤ 40 cm), and over-mature trees (DBH > 40 cm).

The age structure dynamic change index (*V_n_, %*) of the *P. euphratica* population between two distinct age structures was calculated using the following formula [30,36]:

(1) $$V_{n}=\frac{S_{n}-S_{n+1}}{max\;(S_{n}\ldots S_{n+1})}\times 100$$

where *S_n_* and *S_n+1_* were the number of trees in the *n* and *n + 1* age structure, respectively; max (……) was the maximum value in the brackets; and *V_n_* values ranged between −1 and 1. A positive, negative, or zero *V_n_* value indicated growth, decline, or stability in the population structure between adjacent age classes. The overall population age structure dynamic change index (*V_pi_, %*) was calculated using the following formula [30,36]:

(2) $$V_{pi}=\frac{1}{\sum_{n=1}^{k-1}S_{n}}\sum_{n=1}^{k-1}\left(S_{n}\times V_{n}\right)$$

Similar to *V_n_*, positive, negative, and zero values of *V_pi_* signified growth, decline, or stability in the entire population’s age structure. *k* represented the largest age class in the study population.

A survival curve of the population was constructed using age class as the X-axis and ln (*lx*) as the Y-axis [30]. Male and female individuals were distinguished based on their distinct flowers. The operational sex ratio (*OSR*) was determined as the male-to-female (M/F) number ratio, and the chi-square test was used to assess significant deviation from a 1:1 ratio.

### 2.4. Measurement of Annual Growth Rate

In October 2015, we randomly selected 1–3 male and female trees every 50 m along the plant survey transect to collect core samples for evaluating annual tree growth. Sample trees of the same sex were spaced farther apart, with a straight-line distance of more than 20 m between them, separated by trees of different sexes. Two cores were collected from each tree at breast height using a 10 mm increment borer (Haglöf increment borer, Sweden). In total, 96 tree ring samples were cored. Following air-drying, mounting, and sanding, all tree cores were measured using a Lintab ring-width measuring system with 0.001 mm accuracy. Cross-dating quality and accuracy were controlled through the Cofecha program. The shared time period for all cores was 1976–2015.

The annual growth rate of tree rings (*R*, %) was calculated using the following formula [37]:

(3) $$R=\frac{r_{i}}{\sum_{n}^{i}r}\times 100$$

where *r_i_* was the annual tree ring width (mm); $\sum_{n}^{i}r$ was the sum of tree ring widths (mm).

The percentage of tree ring growth change (*G, %*) was calculated using the following formula [37]:

(4) $$G=\frac{G_{n+1}-G_{n}}{G_{n}}\times 100$$

where *G_n_* and *G_n+1_* were the mean tree ring width of the first five years and the subsequent five years, respectively. If G was more than 25%, 50%, or 100% for 5–10 years, the trees exhibited a distinct growth release process, and if G was less than −25%, −50%, or −100% for 5–10 years, the trees exhibited a distinct growth inhibition process. Mild, moderate, and severe release or inhibition were identified when G exceeded ±25%, ±50%, or ±100%, respectively [37]. The year corresponding to the maximum or minimum value in each growth change percentage sequence marked the release or inhibition year.

### 2.5. Measurement of Intrinsic Water Use Efficiency

We extracted annual tree rings from the cores using a razor blade spanning the entire growth year of each ring. To avoid contamination, we excluded the sanded and glued surfaces of the rings. As tree rings in some years are very narrow, making single tree ring samples insufficient for measurement, we created mixed samples of the same gender cores for each year. These mixed samples were formed by combining equal amounts of the same-year tree cores of all cores with the same sex. Subsequently, mixed samples of female and male annual rings were collected for each year between 1975 and 2015. These mixed samples were then pulverized into powder.

A stable isotope mass spectrometry system (Elementar Isoprime 100, UK) was employed to analyze the stable carbon isotope (*δ^13^C*) of the tree ring powder (2–5 mg per sample). The data of *δ^13^C* (‰) were calculated using the following formula [38]:

(5) $$\delta^{13}C=(\frac{R_{sample}}{R_{standard}}-1)\times 1000$$

where *R_sample_* and *R_standard_* were the ^13^C/^12^C ratios in the sample and standard, respectively. The analytical system error was less than ≤0.2‰.

*WUE_i_*, reflecting the ratio of carbon uptake to stomatal conductance, was measured using stable carbon isotope ratios (*δ^13^C*) [14]. *WUE_i_* (µmol CO_2_·mol^−1^ H_2_O) was evaluated based on *δ^13^C* using the following formula [31,39,40]:

(6) $${∆}^{13}C=\frac{\delta^{13}C_{a}-\delta^{13}C}{1+\delta^{13}C}\;$$

(7) $${WUE}_{i}=\frac{C_{a}\times (b-{∆}^{13}C)}{1.6\times (b-a)}$$

where ${∆}^{13}C\;was$ the discrimination value of stable carbon isotopes in tree rings; *a*, 4.4‰, was the diffusion fractionation coefficient of CO_2_ passing through pores; *b*, 27‰, was the fractionation coefficient of CO_2_ during carboxylation by the Rubisco enzyme; *C_a_* (µmol·mol^−1^) was the atmospheric CO_2_ concentration in the study area; and *δ^13^C* and *δ^13^C_a_* were the carbon isotope ratios of tree rings and atmosphere, respectively.

### 2.6. Statistical Analysis

Curve estimation was used to analyze the relation between groundwater depths and the distances far from the river. An analysis of variance (ANOVA) was conducted to assess differences between male and female *P. euphratica* for measured parameters, while Pearson and Spearman’s correlations examined the relationships between WUEi, annual growth rates, and groundwater depths. The data satisfied ANOVA assumptions. Tukey–Kramer analysis aided multiple comparisons. Statistical analyses were performed using SPSS 13.0 (SPSS Inc., Chicago, IL, USA), and figure creation employed Sigmaplot 12.5 (Systat Software, Inc., San Jose, CA, USA) [22].

## 3. Results

### 3.1. Population Characteristics of P. euphratica under Different Groundwater Depths

**Figure 3:**
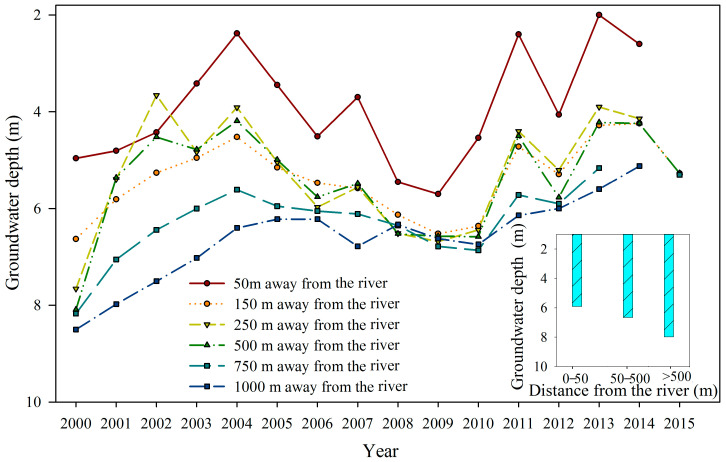
Groundwater depth changes in the Yingsu hydrological monitoring section in the lower Tarim River, China from 2000 to 2015.

Field investigation data from 2000 to 2015 revealed fluctuations in groundwater depth relative to the distance from the river (Figure 3; y = 2 × 10^−6^x^2^ – 0.0041x + 831.71, R^2^ = 0.9966). Moreover, in 2000, the groundwater depths within 50 m, 50–500 m, and 500–1000 m from the river were less than 6 m, 6–7 m, and more than 7 m, respectively (Figure 3), indicating an increase in groundwater depth with greater distance from the river.

#### 3.1.1. Age Structure Characteristics of *P. euphratica*

**Figure 4:**
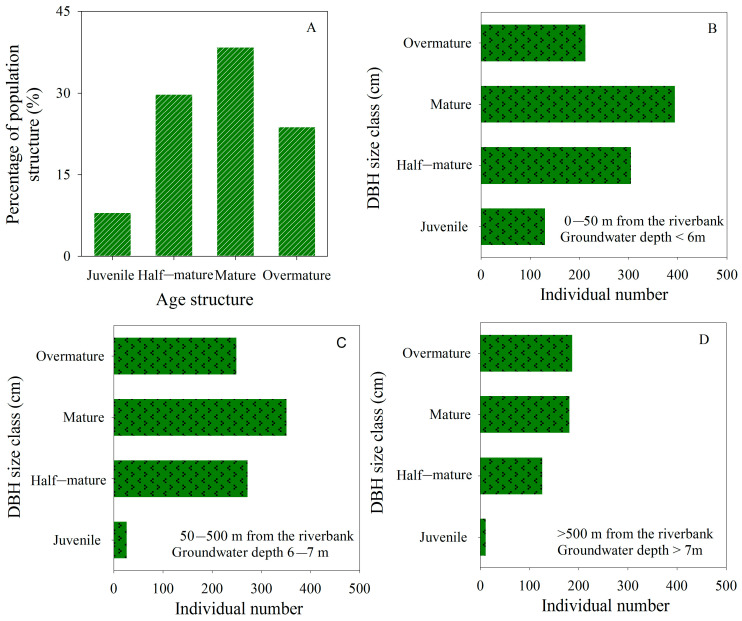
Age structure percentage of *Populus euphratica* population (**A**), the number of diameters at breast height (DBH) and size class of natural *Populus euphratica* in 0–50 m (**B**), 50–500 m (**C**), and >500 m (**D**) from the riverbank in the lower Tarim River, China.

*P. euphratica* was sparsely distributed in the lower reaches of the Tarim River. Within 1000 m of the river channel, the average density of *P euphratica* trees was 0.01 plants/m^2^. Young, half-mature, mature, and over-mature trees constituted 8.05%, 29.76%, 38.42%, and 23.78% of the tree population in the transect, respectively (Figure 4A). Field observation indicated that young trees primarily inhabited the area within 50 m of the river channel, where groundwater depth was less than 6 m (Figure 4B–D). Half-mature and mature trees exhibited a relatively even distribution within 500 m from the river channel, where groundwater depth ranged from 6 to 7 m (Figure 4B,C). Beyond 500 m from the river channel, where groundwater depth exceeded 7 m, the dominant type was mature and over-mature trees (Figure 4D). This observation suggested that the regeneration capacity of the *P. euphratica* population weakened with increasing groundwater depth.

**Figure 5:**
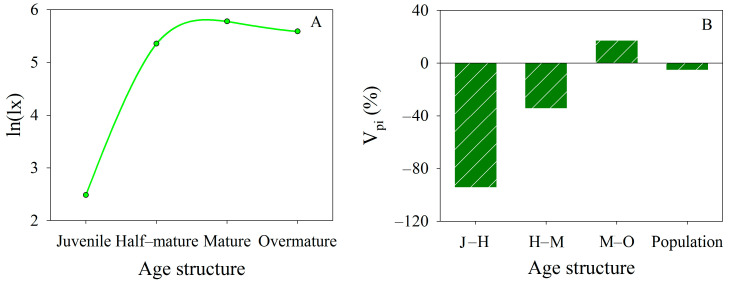
Survival curves (*ln(lx),* **A**) and age structure dynamic change indices (*V_pi_*, **B**) of *P. euphratica* in the lower Tarim River, China.

*Lx* also demonstrated variations with tree age. The survival ratio was lowest for young trees, gradually increasing with age until stabilizing in the mature and over-mature stages (Figure 5A). *V_pi_* for the *P. euphratica* population across different age classes were negative (Figure 5B), indicating a declining trend in the transitions from young to half-mature, half-mature to mature, and mature to over-mature trees. These findings showed a clear trend of aging and decline within the *P. euphratica* population in the lower Tarim River. Moreover, in conjunction with the spatial distribution, the degree of aging and decline of the *P. euphratica* population increased with rising groundwater depth.

#### 3.1.2. Operational Sex Ratio (*OSR*) of *P. euphratica*

**Figure 6:**
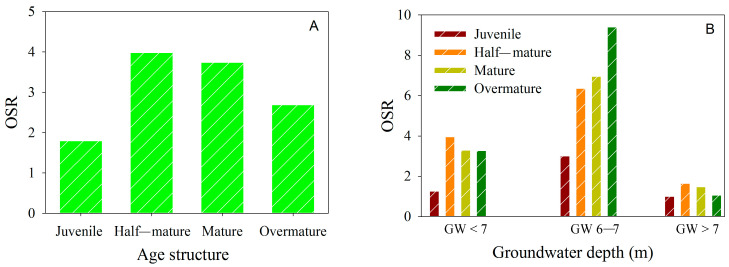
Changes in operational sex ratio (*OSR*) with age (**A**); changes in *OSR* with groundwater depth (**B**) of natural *Populus euphratica* population.

The *OSR* of *P. euphratica* also displayed variations with tree age (Figure 6A). *OSR* was 1.80:1 in young *P. euphratica* communities, increasing to approximately 4:1 in half-mature communities, indicating male tree dominance. However, the *OSR* gradually declined with age, reaching 3.74:1 and 2.68:1 in the mature and over-mature *P. euphratica* community, respectively.

While *P. euphratica* naturally exhibits male bias within the riparian forest community, the *OSR* of *P. euphratica* varied with changing groundwater depth (Figure 6B). Specifically, the average *OSR* for *P. euphratica* was 2.94 when groundwater depth was less than 6 m, increasing to 6.43 for groundwater depth of 6–7 m and decreasing to 1.29 for groundwater depth exceeding 7 m. These results indicated that male *P. euphratica* held an advantage, particularly when groundwater depth was less than 7m. However, this advantage diminished significantly as groundwater depth exceeded 7 m, and male dominance within the community decreased.

### 3.2. Long-Term Growth Difference between Male and Female P. euphratica under Different Groundwater Depths

**Figure 7:**
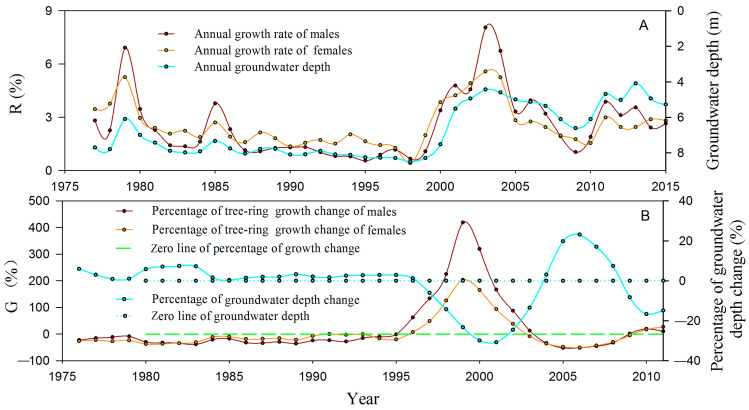
Annual growth percentage (*R*, **A**) and percentage of tree ring growth change (*G,* **B**) in natural *Populus euphratica* under different groundwater depths in the lower Tarim River, China.

The *R* of *P. euphratica* exhibited a close correlation with groundwater depth (*R_male_* = −0.656, *R_female_* = −0.779; *p* < 0.01; *n* = 40) based on data from 1976 to 2015. During the period of 1976–1999, when the groundwater depth exceeded 7 m (Figure 7A), its fluctuations remained relatively stable (Figure 7B). *R* for male and female *P. euphratica* were recorded as 1.80% and 2.14%, respectively (Figure 7A). *R* exhibited negative change rates of −7.90% and −6.69% for males and females, respectively (Figure 7B), during this period. This indicated a gradual decline in the annual growth rate of *P. euphratica* for both sexes when groundwater depth exceeded 7 m. Notably, no significant distinction in *R* between males and females was observed (Figure 7A, *p* > 0.01; *n* = 48).

Conversely, during 2000–2015, the *R* of both male and female *P. euphratica* exhibited notably rapid responses to the pronounced fluctuations in groundwater depth (Figure 7). From 2000 to 2015, the average *R* values were 3.66% for males and 3.17% for females, with change rates of 9.86% and 5.20%, respectively. Compared to 1976–1999, the average *R* for *P. euphratica* increased remarkably by 129.55% in 2000–2015. Moreover, the mean *R* of males and females increased by 184.83% and 89.11%, respectively. This result showed a sharp increase in *R* of *P. euphratica* when the groundwater depth decreased to less than 7 m. Furthermore, the increase in *R* was significantly greater in males than in females (Figure 7A; *p* < 0.05; *n* = 32).

The *G* of *P. euphratica* also exhibited a significant negative correlation with groundwater depth (Figure 7B; *R_male_* = −0.591, *R_female_* = −0.571; *p* < 0.01; *n* = 40). In 1975–1999, the average *G* was −25.40% for males and −17.65% for females (Figure 7B), suggesting a moderate inhibition for male *P. euphratica* growth and a mild inhibition for female *P. euphratica* growth. However, the observed inhibitions in growth did not display a significant distinction between males and females when the groundwater depth exceeded 7 m (Figure 7B; *p* > 0.01; *n* = 48). Conversely, when the groundwater depth underwent rapid fluctuations and decreased to less than 7 m from 2000 to 2015, the average *G* was recorded as 77.64% for male *P. euphratica*, with a variation range of 62.16–418.79%, indicating a severe release. For female *P. euphratica*, the average *G* was 31.63%, with a variation range of 7.38–203.34%%, showing a moderate release. Although the key release year of growth both for females and males occurred in 2000, the degree of growth release in males was significantly higher than that in females (Figure 7B, *p* < 0.01; *n* = 32).

### 3.3. Long-Term Water Use Efficiency Differences between Male and Female P. euphratica under Different Groundwater Depths

**Figure 8:**
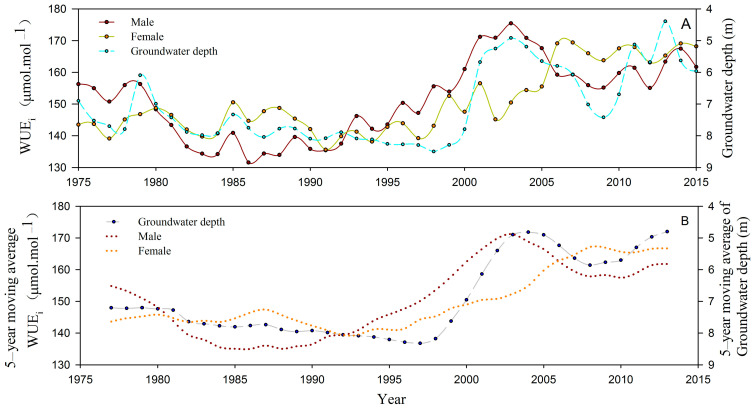
*WUE_i_* (**A**) and 5—year moving average *WUE_i_* (**B**) of natural *Populus euphratica* under different groundwater depths in the lower Tarim River, China.

The *WUE_i_* of *P. euphratica* in both males and females showed significant increases with decreasing groundwater depth (Figure 8A). The *WUE_i_* of female *P. euphratica* remained relatively stable at an average of 143.55 ± 2.22 µmol CO_2_·mol^−1^ H_2_O with an annual change rate of 0.30% when the groundwater depth was more than 7 m and increased significantly to an average of 159.40 ± 7.58 µmol CO_2_·mol^−1^ H_2_O with an annual change rate of 0.68% under groundwater depths less than 7 m from 2000. Male *P. euphratica* exhibited a significantly linear, negative correlation between *WUE_i_* and groundwater depth (*R* = −0.803, *p* < 0.01; *n* = 40), and the *WUE_i_* significantly rose from 142.61 ± 6.86 µmol CO_2_·mol^−1^ H_2_O to 162.72 ± 4.72 µmol CO_2_·mol^−1^ H_2_O under groundwater depths less than 7 m from 2000.

ANOVA analysis showed that the *WUE_i_* of male *P. euphratica* was significantly higher than that of the female *P. euphratica* when the groundwater depth ranged from 6 to 7 m (Figure 8; *p* < 0.01; *n* = 10). Conversely, the *WUE_i_* of female *P. euphratica* was significantly higher than that of males when the groundwater depth exceeded 7 m (Figure 8; *p* < 0.01; *n* = 48), as well as when it was less than 6 m (Figure 8B; *p* < 0.05; *n* = 22). Therefore, compared to females, the *WUE_i_* of male *P. euphratica* exhibited heightened sensitivity and a quicker response to the changes in groundwater depth.

## 4. Discussion

### 4.1. Effects of Groundwater Depth Change Caused by Ecological Water Conveyance on P. euphratica Forest Restoration

**Figure 9:**
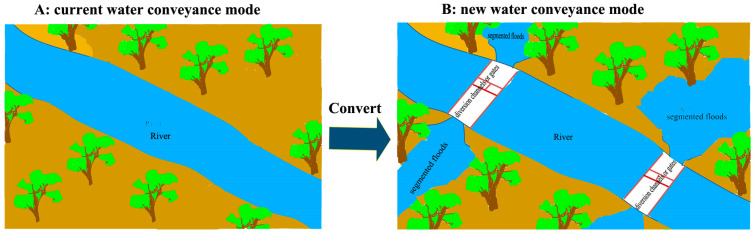
Sketch of current ecological water conveyance mode (**A**) and optimized ecological water conveyance mode with segmented floods (**B**).

The EWCP has been implemented for more than 20 years in the lower Tarim River. The ecological water was conveyed along a fixed river channel, with uncertain water quantities and delivery timings each year (Figure 9). In the lower Tarim River, the EWCP has significantly decreased groundwater depth through groundwater recharge, decreasing from over 8 m in 2000 to less than 6 m in 2015 (Figure 2), but the groundwater depth has a significantly positive correlation with the distance from the river (R^2^ = 0.9966, Figure 3) [34]. Several researchers have confirmed that *P. euphratica* can grow normally under groundwater depths of less than 6 m, experience mild drought stress between groundwater depths of 6–7 m and suffer severe drought stress when the groundwater depth exceeds 7 m [25,29,41]. Similarly, our investigation into the *P. euphratica* population revealed that young individuals, constituting less than 2% of all trees in the natural population, primarily grew within 50 m of the river, where the groundwater depth was less than 6 m (Figure 4). The number of *P. euphratica* individuals gradually decreased as groundwater depth increased, and a pronounced reduction in juvenile, half-mature, and mature trees was observed when groundwater depth exceeded 7 m (Figure 4). This suggested that the regeneration capacity and sustainability of *P. euphratica* weakened when groundwater depth exceeded 6 m.

Although researchers observed that the EWCP increased vegetation coverage area in the desert riparian *P. euphratica* forest from 492 km^2^ to 1423 km^2^ and from 2000 to 2020 [20], and also increased the NPP of natural vegetation with an average rate of 0.40 g C∙m^−2^ ∙a^−1^ from 2001 to 2019 [42], these positive trends in coverage and NPP diminished as the distance from the river increased [20,42]. The most clear changes in coverage and NPP only occurred where groundwater depth was less than 6 m [20,42]. Consequently, altering the aging and declining patterns of the *P. euphratica* population is challenging, which is a fact supported by both survival curves and dynamic indices of the *P. euphratica* population (Figure 5).

### 4.2. Causes of Sexual Spatial Segregation between Male and Female P. euphratica

Research has shown that dioecious plants exhibited sexual dimorphism in eco-physiological traits when subjected to changing environmental factors [7,17,43,44,45]. For *P. euphratica*, no significant sexual differences in eco-physiological traits between males and females were observed under well-watered conditions [2]. However, under mild drought stress, male *P. euphratica* exhibited significantly higher biomass, stomatal density, stomatal length, net photosynthetic rate, and photosynthetic electron transfer rate (ETR) compared to females [2,46]. Our study also identified that *R* and *WUE_i_* of male *P. euphratica* were more responsive to changes in groundwater depth compared to females. Furthermore, the *R* and *WUE_i_* of males were significantly higher than those of females under mild drought stress, where the groundwater depth ranged from 6 to 7 m (Figure 7 and Figure 8). This phenomenon could be attributed to the higher developmental cost of females in dioecious plants, as well as the competition between female reproduction and growth when water resources are limited [7,8]. Female *P. euphratica* allocated more photosynthesis and organic matter to reproduction, while males allocated more photosynthesis and organic matter to growth, thereby facilitating their dominance in the community. These findings might provide an explanation for the observed highest *OSR* at groundwater depths of 6–7 m (Figure 6).

In contrast, females exhibited higher levels of malondialdehyde (MDA), antioxidant enzyme activity, proline content, soluble sugar content, and relative water content of leaves compared to males under severe drought stress [46]. Our study similarly observed that the *WUE_i_* of females was significantly higher than that of males when *P. euphratica* suffered severe drought stress (Figure 7 and Figure 8). These findings suggest that female *P. euphratica* possessed a broader range of drought tolerance compared to males due to their greater regulatory capacity in terms of biochemistry and water use efficiency during severe drought stress. To elaborate, when a significant reduction in groundwater depth led to mild drought stress, male *P. euphratica* experienced rapid growth along with heightened *WUE_i_*, resulting in a faster recovery and earlier resurgence of males compared to females. However, when a substantial increase in groundwater depth led to severe drought stress, the growth advantages of male *P. euphratica* diminished significantly due to lower *WUE_i,_* potentially resulting in male decline or mortality before females. Based on these findings, it is plausible that female *P. euphratica* exhibited a higher survival probability than males under extreme drought stress, thus explaining the significant decrease in OSR when groundwater depth exceeded 7 m (Figure 6). Consequently, the divergent *WUE_i_* responses of males and females to drought stress underlie the sexual spatial segregation of *P. euphratica* in the lower reaches of the Tarim River Basin.

### 4.3. Optimized Ecological Water Conveyance Mode for Future Restoration and Regeneration of P. euphratica Forest

Usually, insufficient numbers of either males or females may hinder the population’s sexual reproduction, impeding succession and renewal. In the lower Tarim River, *P. euphratica* displayed male bias within the natural riparian forest (Figure 6). *P. euphratica* employed sexual reproduction and asexual reproduction modes. However, due to the low seed vitality and a ripening period extending up to 141 days [47], the germination of *P. euphratica* seeds was hard due to limited surface water resources. Therefore, the sexual reproduction of *P. euphratica* was hindered, and the asexual reproduction of roots became preferred [20,48], and the horizontal distance of the root sprouts could be up to 40 m [49], which could further intensify the deviation in the sex ratio of the *P. euphratica* population. Nevertheless, root asexual reproduction could reduce hybridization and gene diversity, compromising population adaptability and exacerbating the degradation of the population [20]. Moreover, some studies showed that declining groundwater should lower the asexual reproduction of roots even more than that of generatively grown trees [49]. Additionally, juvenile *P. euphratica* is primarily concentrated in areas with a groundwater depth of less than 6 m (Figure 4). Furthermore, populations sharply declined in areas with groundwater depths exceeding 7 m (Figure 4), where both sexual and asexual reproduction were hard, leading to further population decline and mortality. Based on these findings, although the existing EWCP mode, in operation for over 20 years, improves the ecological environment, it also struggles to effectively regenerate and fundamentally reverse the degradation of the *P. euphratica* population in the lower Tarim River (Figure 5). In particular, the aging and decline of the *P. euphratica* population could escalate with deeper groundwater depths. Consequently, the pivotal challenge for the current ecological water conveyance mode is how to stimulate seed germination or root asexual reproduction to establish young individuals for stable and healthy population succession.

Our findings highlight that the renewal and regeneration of the *P. euphratica* population are most likely within areas with groundwater depths of less than 6 m (Figure 4 and Figure 5). Moreover, wet surface soil post-flood facilitates seed and root asexual reproduction [20,47], elucidating the scarcity of young trees beyond 50 m from the river. Furthermore, the study suggested that flooding was more favorable to generative than clonal reproduction [48]. The existing ecological water conveyance project (EWCP), with uncertain quantities and timings along a fixed and single channel, recharged groundwater via lateral seepage (Figure 9A), resulting in groundwater recharge positively correlated to distance from the river (R^2^ = 0.9966) [34]. As a result, surface soil flooding is challenging under the current EWCP mode. Furthermore, the limited lateral groundwater reach of linear rivers yields substantial groundwater depth variation gradients along distances from the river [34]. These factors exacerbated sexual spatial segregation within the *P. euphratica* population, jeopardizing its future vitality, regeneration, and sustainability.

Addressing these concerns necessitates updating the ecological water conveyance mode. For instance, water diversion channels or sluice gates could be installed in hydrological monitoring sections along river channels, enabling future ecological water conveyance during the annual breeding season via these channels or gates (Figure 9B). This update could rapidly expand the lateral seepage and inundation areas for recharged groundwater both alongside and away from the river, fostering widespread seed and root reproduction to form new seedlings at a large scale. Moreover, by broadening flood areas under the new EWCP mode, the seeds of female *P. euphratica* might germinate to generate new female or male seedlings with equal probability [50], potentially equalizing the frequency of females and, thus, reducing the *OSR* of the *P. euphratica* population. Consequently, this new ecological water conveyance approach could enhance the age structure of the *P. euphratica* population and facilitate effective restoration and optimal succession of the *P. euphratica* forest.

## 5. Conclusions

In summary, the riparian *P. euphratica* forest in the lower reaches of the Tarim River exhibited signs of aging and degradation due to long-term drought stress resulting from changes in groundwater depth. This trend intensified with increasing groundwater depth. Although *P. euphratica* is a male-biased species within the natural riparian forest, the *OSR* of *P. euphratica* varied in response to the degree of drought stress. The highest *OSR* for *P. euphratica* was observed under mild drought stress, while it decreased under non-drought and severe drought stress conditions. Male *P. euphratica* demonstrated a competitive advantage characterized by a higher growth rate and *WUE_i_* under mild drought stress, enhancing their survival competitiveness within the population. However, this competitive advantage sharply diminished under severe drought stress, accompanied by lower *WUE_i_*. In contrast, female *P. euphratica* exhibited higher *WUE_i_* than males under both severe drought and non-drought stress, resulting in a broader drought stress tolerance range for females. The sexual responses of *P. euphratica* to various drought stresses revealed the changes in the sex ratio and spatial segregation of males and females in the lower Tarim River. These findings shed new light on the implications of ecological water conveyance management for the restoration, conservation, and rejuvenation of the natural *P. euphratica* forest.

## Data Availability

The data can be obtained by contacting the author by email.
